# An American Clinical Training Program for Spanish Nutrition Support Pharmacists: A Three-Year Experience

**DOI:** 10.3390/pharmacy3010003

**Published:** 2015-02-25

**Authors:** Roland N. Dickerson, Eva M. Martinez, M. Carmen Fraile, Josefina Giménez, M. Victoria Calvo

**Affiliations:** 1 Department of Clinical Pharmacy, University of Tennessee College of Pharmacy, 881 Madison Avenue, Suite 345, Memphis, TN 38163, USA; 2 Pharmacy Service, Corporació de Salut del Maresme i la Selva, Sant Jaume, 209-217, 08370 Calella, Barcelona, Spain; E-Mail: emartinezbernabe@salutms.cat; 3 Department of Pharmacy, University Hospital of Candelaria, Crta. Rosario No 145, 38010 Santa Cruz de Tenerife, Spain; E-Mail: cfracle@gobiernodecanarias.org; 4 Department of Pharmacy, Manacor Hospital, Ctra Manacor-Alcudia, 07500 Manacor, Balearic Islands, Spain; E-Mail: jgimenez@hmanacor.org; 5 Department of Pharmacy, University Hospital of Salamanca, Paseo de San Vicente, 88, 37007 Salamanca, Spain; E-Mail: toyi@usal.es

**Keywords:** parenteral nutrition, enteral nutrition, pharmacist, clinical practice, board certification, education, nutrition, nutrition support

## Abstract

A clinical nutrition support pharmacist training program, in collaboration with the Spanish Foundation of Hospital Pharmacy, Spanish Society of Clinical Nutrition, Abbott Nutrition International, University of Tennessee, College of Pharmacy and Regional One Health, is described. Nutrition support pharmacists from Spain were selected to participate in a one-month training program with an experienced board-certified nutrition support pharmacist faculty member within an interdisciplinary nutrition support team environment in the U.S. Participants were expected to actively engage in an advanced clinical practice role with supervision. Clinical activities included daily intensive patient monitoring, physical assessment, critical evaluation of the patient and development of an appropriate treatment plan for patients receiving either enteral or parenteral nutrition therapy. Upon successful completion of the training program, participants were anticipated to incorporate these techniques into their current practice in Spain and to train other pharmacists to function in an advanced clinical role independently or within an interdisciplinary nutrition support team environment.

## 1. Introduction

The contributions of nutrition support teams are well established [1,2,3,4]. Pharmacists have been intimately involved in nutrition support since the inception of these teams, especially regarding the provision of parenteral nutrition [4]. The first documentation of the role of a clinical nutrition support pharmacist within an interdisciplinary team was published four decades ago [4]. Although formalized education and training in nutrition support for pharmacists in collaboration with universities and hospitals has existed for decades in the U.S. [5], opportunities in Spain have been primarily limited to residency training and post-residency self-learning activities. Because of a high level of interest and commitment among numerous self-directed Spanish clinical pharmacists, Spain is second in the number of countries with board-certified nutrition support pharmacists (BCNSP) world-wide [6]. Many Spanish pharmacy nutrition support pharmacists with an advanced clinical practice must practice independently due to the lack of an interdisciplinary clinical service that evaluates and treats patients collectively as a team on a routine basis. In addition, clinical privileges for most Spanish nutrition support pharmacists are often directed solely for parenteral nutrition.

The nutrition support pharmacist training program for international pharmacists at the University of Tennessee, College of Pharmacy and Regional One Health (formerly Regional Medical Center at Memphis) provided the opportunity for pharmacists to actively participate within an interdisciplinary nutrition support service (NSS). The Spanish trainees were expected to refine their intensive monitoring skills, provide a concise verbal patient presentation to the team, complete a written daily clinical progress note [7] and verbally interact with non-NSS healthcare professionals (e.g., unit nurses, speech therapists, primary service physicians, *etc.*). In addition, Spanish pharmacist trainees were expected to develop the skills necessary for the appropriate management of patients receiving either enteral or parenteral nutrition. The authors describe an advanced experiential practice program developed for nutrition support pharmacists and the impact of this clinical training program on their practice at their respective home institutions in Spain.

## 2. Development and Administration of the Program

The University of Tennessee, College of Pharmacy, in conjunction with Regional One Health (formerly Regional Medical Center at Memphis), was identified by the senior members of the Spanish Nutrition Support Pharmacists group as the preferred site for clinical training of its nutrition support scholarship recipients. Dr. Roland N. Dickerson served as the mentor for the international trainees. The NSS is a consult and manage service, whereby once the service is consulted, the nutrition team has clinical privileges for prescribing parenteral and enteral nutrition, management of fluid and electrolytes, glycemic control, acid-base disorder management, vitamins and minerals, pharmacotherapy related to nutrition support therapy (e.g., prokinetic agents, anti-diarrheal medications, laxatives, avoidance of drug-nutrient interactions, *etc.*) and ordering of follow-up laboratories. Close communication between the NSS and patient’s primary service is mandatory to avoid any potential overlap, omissions or errors in the patient’s therapy. Only the NSS at Regional One Health can prescribe parenteral nutrition therapy, whereas the primary medical or surgical services have the option to manage patients requiring enteral nutrition therapy themselves or to consult the NSS, depending on the patient’s acuity, complexity and comfort level and on the primary service’s ability to effectively and safely manage the patient.

A previously established monthly experiential training program for pharmacy students at the University of Tennessee, College of Pharmacy and Regional One Health served as the platform for the training of the Spanish pharmacists. The Spanish pharmacists were incorporated into the College’s international pharmacy student exchange program and were allowed the same rights of a student of the University of Tennessee, College of Pharmacy.

A call for applications to Spanish nutrition support pharmacists interested in the training program occurred a few months before selection of the candidate. Oversight of the program’s application and selection process was directed by Dr. M. Victoria Calvo and administered by members of the Nutrition Support Pharmacists group of the Spanish Society of Hospital Pharmacy. Successful candidates must have already successfully completed a hospital pharmacy residency. Multiple years of documented practice experience in pharmacy nutrition support and board certification in nutrition support pharmacy (BCNSP) from the Board of Pharmacy Specialties were desirable pre-requisites for the successful candidate. Demonstration of proficiency in verbal and written communication in the English language via a standardized testing service was mandatory.

Funding for the month-long training scholarship was provided by Abbott Nutrition Spain via an unrestricted educational grant to the Spanish Society of Hospital Pharmacy Foundation. Six thousand euros were provided to the successful candidate to assist with the expenses of rent, food, flight, car rental and J-1 visa application. One candidate was selected yearly from the pool of applicants to participate in this training program. The University of Tennessee, College of Pharmacy and the faculty preceptor voluntarily participated in this program without funding. It was anticipated that this program would potentially lead to future sites for the College’s advanced pharmacy practice experience program for an international pharmacy rotation for its students, expand its reputation for excellence in clinical pharmacy practice, education and research internationally and to support the growth and development of pharmacy nutrition support world-wide.

## 3. Experiential Rotation Objectives and Activities

The learning objectives for the training scholarship program were derived from the pre-existing objectives used in the University of Tennessee, College of Pharmacy curriculum for advanced pharmacy practice experiences (Table 1). More specifically, as related to the nutrition support and advanced practice experience, the trainee must be able to:

(1) Demonstrate the ability to perform and communicate, both verbally and in writing, nutritional and metabolic assessments of the patient.
(2) Write a formal nutrition support progress note and a nutrition support service consult that will become part of the patient’s permanent hospital record.
(3) Develop an individualized nutrition therapy regimen for a patient, including route of nutrition (*i.e.*, enteral *vs.* parenteral), selection or development of the most appropriate formula, the method and rate of administration and identify therapeutic endpoints.
(4) Effectively monitor assigned patients who are receiving nutrition therapy.
(5) Develop a daily plan taking into consideration the patient’s primary diagnosis, secondary diagnoses and clinical conditions, chronic diseases, concurrent drug therapy, laboratory data, fluid balance, glycemic control, acid-base status, physical assessment and any subjective information.
(6) Integrate nutrition support as part of critical care practice and exemplify the role of a clinical pharmacist in the interdisciplinary nutrition support team.

**Table 1:** Learning objectives of the advanced pharmacy practice experience.

As a Result of Successful Completion of the Rotation, the Trainee Will Be Able to:
Communicate well with patients and health professionals Develop, implement, evaluate and modify the pharmaceutical and nutrition care plan Assess the patient and request appropriate medical or laboratory tests Optimize therapy using clinical information Collaborate with other health professionals Evaluate and document interventions and outcomes Think critically and solve problems related to medication and nutrition therapy Make and defend rational and ethical decisions Demonstrate a professional attitude

### 3.1. Scheduled Daily Activities

The trainee was expected to arrive at the hospital approximately at 7 a.m. and leave by 3 or 4 p.m. Monday through Friday. Trainees were assigned to intensively monitor between three to six patients daily. Patients would be either surgical, medical or multiple trauma patients, and most were critically ill, intensive care unit patients. The trainees monitor their patients and write their progress notes during the first 3 h of each day. At approximately 10 a.m., the interdisciplinary team (surgical attending or pharmacy attending, surgical intern, dietitian, pharmacy resident, pharmacy students, international pharmacist trainee) convened at a designated patient’s bedside located in one of the intensive care units to start patient care rounds. The pharmacy trainee actively participated in NSS patient care rounds where all (approximately 20) patients are monitored, evaluated, discussed and the pharmacotherapy/nutrition orders are subsequently written. The trainees were expected to verbally present their monitored patients to the team based on all of the information they previously gathered and to make recommendations. The trainee’s progress notes, including recommendations, were edited as necessary and approved by co-signature of one of the licensed members of the team. A standardized daily monitoring form, which becomes part of the patient’s permanent hospital record, was previously developed to maintain consistency and to ensure a certain level of quality necessary to make informed clinical decisions and plan appropriate therapy. The development of this standardized monitoring form was necessary within an academic-oriented clinical service, as the primary patient monitoring personnel (pharmacy and surgical residents, dietitians and pharmacy students) of the team exhibit significant diversity in experience and training. A copy of the standardized progress note form has been published elsewhere [6]. Like the other members of the team with patient monitoring duties, the Spanish pharmacist trainee completed this documentation prior to clinical rounds and used it as a visual aid during their verbal presentation of the patient on a daily basis. This process generally required approximately an additional 3 to 4 h per day. The international pharmacist trainees were not allowed to write medication, nutrition or laboratory orders with a physician co-signature, as they were not licensed pharmacists in Tennessee and essentially functioned similar to a pharmacy student.

Many clinical pharmacists engaged in nutrition therapy in the U.S. and Spain are focused on only those who require parenteral nutrition therapy. However, at the University of Tennessee and Regional One Health, pharmacists are required to be actively engaged in the monitoring and therapy for patients who require either enteral or parenteral nutrition therapy. Advanced knowledge and practice experience with both parenteral and enteral nutrition therapy is necessary, because critically ill patients may transition from parenteral to enteral nutrition and *vice versa*, depending on enteral feeding tolerance. Therefore, it is important for each discipline to adequately cross-train each other for maintenance of a certain level of functionality of the service when one discipline is temporarily disengaged from patient care rounds. As a result, it is necessary for the pharmacist trainee to learn how to perform a physical exam of the abdomen, interview the patient to ensure tolerance of the enteral feeding and perform a cursory physical exam for the presence of edema, dehydration and nutritional assessment. Development of those skills necessary for planning, initiating and monitoring patients who receive enteral nutrition therapy are required. Basic knowledge of intravenous central and peripheral catheters, drainage tubes and enteral feeding tubes and access sites is mandatory. Thus, knowledge and skills traditionally not in the realm of the pharmacy profession are required in an effort to be a multi-skilled nutrition support health professional to optimize patient care [8]. A synopsis of the minimal expectations for activities required for monitoring patients who receive nutrition therapy is given to all pharmacy students, residents and trainees upon starting the nutrition support advanced pharmacy practice experience (Table 2). It was required that the items in the synopsis guideline were followed for effective evaluation of the patient, writing of the daily progress note and planning therapeutic interventions. Development of these skills was essential for an advanced clinical practice for effectively monitoring the patient and was emphasized throughout the training period.

**Table 2:** Nutrition support monitoring guidelines.

In addition to the collection of the day’s laboratories and volume input/output data, recording of current nutrition and medication therapy, blood glucose concentrations and amount of insulin received, serial gastric residual volumes and number of stools/consistency from the past 24 h, the following must be done:
(1) Review all orders from the past 24 h. Specific items to evaluate include:
(a) Changes in intravenous fluid administration. (b) Electrolyte or insulin administration. (c) New antibiotics ordered for infections. (d) New drugs ordered or discontinued that may affect electrolyte balance, result in diarrhea or constipation, decrease appetite, worsen glycemic control or alter energy/protein requirements. (e) Pre-operative orders for a surgical procedure. (f) New orders for vasoactive drugs. (g) Changes in dietary orders (e.g., *nil-per-os* to a liquid diet) (h) Changes in respiratory status or mechanical ventilation settings.
(2) Read all progress notes from the past 24 h. Specific items to evaluate include:
(a) Overall change in patient’s clinical status. (b) Any new diagnoses or complications. (c) Results of any radiology or interventional studies. (d) Any concerns expressed about nutrition, electrolyte balance, glycemic control, diarrhea, drainage output or fluid status. (e) Description of abdominal assessment for patients receiving or about to receive enteral nutrition. (f) Operative notes: identify what procedure was done and assess its impact on the current nutrition therapy.
(3) Patient observation. Specific items to evaluate include:
(a) Ask open-ended questions if the patient can converse with you. (b) Ensure that the correct nutrient formula is hanging. (c) Ensure pump settings for the nutrient formula and intravenous fluids are correct. (d) Assess the patient for edema or dehydration. (e) Perform a physical exam of the abdomen. (f) Interview the patient for any subjective complaints or general well-being.
(4) Items that are occasionally overlooked by the trainee include:
(a) Changes in body weight over time attributed to fluid perturbations. (b) Laboratory values that are pertinent for individual patients that are not part of the standardized monitoring form (e.g., thyroid function tests for a patient receiving concurrent enteral nutrition and levothyroxine)
(5) Assess all patient data.
(a) Use judgment as to the most important items above that should be discussed during rounds. (b) Pay special attention to patients who are receiving non-standard amounts (void or excessive) of electrolytes in their parenteral nutrition formulation. (c) After exhausting all available laboratory, clinical and medical chart resources, ask the patient’s nurse for any additional necessary clarifications.
(6) When verbally presenting patients during patient care rounds, present the data in the following order:
(a) A short summary of the patient’s hospital course during the last 24 h (or past 72 h if the patient is to be presented on Monday). (b) Using the standardized progress note, and highlight the major issues. (c) Provide an assessment reflective of all subjective and objective data. (d) Give a detailed therapeutic plan. (e) List those laboratory tests desired for the next day.

### 3.2. Progress Note Evaluation

The previously described standardized progress note also serves as an outcome-focused training experience with inherent capabilities for structured evaluation and assessment of the trainee’s progress. The mentor (RND) randomly chose a patient progress note from the trainee each week for evaluation. Sections of the note were weighed and scored according to a detailed rubric system according to the accuracy and completeness of the retrieval of pertinent information. Details of the progress note and the rubrics for evaluation have been previously published [7]. The required information for the progress note was outlined in a written document describing the specific elements that should be contained in each subsection for the trainee. These elements follow the pattern of a typical “SOAP” (subjective, objective, assessment, plan) progress note, which also included elements specific to nutrition therapy, such as nutrition regimen, intravenous fluids, pertinent pharmacotherapy, fluid intake and output, glycemic control, gastric residuals and abdominal assessment, body temperature/hemodynamic measurements/intracranial pressure, laboratories (e.g., serum chemistries, arterial blood gas measurements, hematology, microbiologic or unique laboratories that pertain to the overall metabolic care), creatinine clearance and nitrogen balance. The mentor reviewed the progress note in conjunction with the evaluation form with the trainee and provided suggestions on how the trainee may improve their performance.

### 3.3. Assigned Readings

Students are given weekly reading assignments of about five articles weekly encompassing the principles to be learned during the nutrition support rotation. These readings are updated on a yearly basis as new information is available and incorporated into our evidence-based nutrition support practice. The students present and discuss these papers on a weekly basis. The Spanish pharmacist trainees were given these papers to be read prior to starting their advanced practice experience. However, most of the articles were already well known to the Spanish pharmacist trainees, as they are commonly used papers in current practice, such as clinical practice guidelines, review articles and key papers supporting current practice. As a result, the Spanish pharmacist trainee did not routinely participate in a weekly review of these basic articles, but were occasionally given primary literature unfamiliar to the trainee regarding management of a particular patient problem encountered during clinical work rounds and discussed thereafter with the mentor.

### 3.4. Other Activities

Trainees were expected to attend our twice monthly Nutrition Support/Critical Care journal club meeting. They were also offered the opportunity to observe access procedures related to nutrition support therapy (*i.e.*, percutaneous endoscopic gastrostomy, ultrasound-guided peripherally-inserted central venous catheter placement and nasal bridle placement), attend the monthly Department of Clinical Pharmacy grand rounds, quarterly Nutrition Support grand rounds and weekly Surgery Grand Round seminars, depending on the availability of and interest to the trainee.

### 3.5. Other Tangible Benefits by Observation of Their Mentor

The traineeship additionally offered the opportunity for the Spanish pharmacist to observe their mentor serve as an interdisciplinary educator for current and future pharmacists, dietitians, nurses and physicians and as a principal investigator for practice-based research initiatives designed to improve patient care. Many past practice-based research initiatives have come to fruition via publication and implementation within the daily clinical practice of the NSS at Regional One Health [9]. Some of the required readings for the advanced pharmacy practice experience to justify the specific management employed by the NSS for frequently encountered problems in daily clinical practice, including fluid and electrolyte management, use of prokinetic therapy, glycemic control with insulin therapy, drug-nutrient interactions and estimation of energy and protein requirements, are directly due to those practice-based research efforts [9].

### 3.6. Impact of Experiential Program

Three nutrition support pharmacists from Spain (Eva Martinez, M. Carmen Fraile and Josefina Giménez) successfully completed the program. Trainees described the following changes in their clinical practice, educational activities and professional endeavors following completion of the experiential training program.

Clinical practice: A common area of expansion was involvement in enteral nutrition therapy, particularly for those patients who were undergoing transitional therapy from parenteral to enteral nutrition therapy. Reported activities for enteral therapy by the former trainees also included involvement in the selection of an appropriate enteral formula for a given patient, provision of pharmacotherapy for improvement of enteral feeding tolerance (e.g., prokinetic therapy and laxatives), management of drug nutrient interactions and involvement in the management of electrolyte disorders for patients who receive enteral nutrition therapy. One trainee reported her active involvement with the Pharmacy and Therapeutics Committee for the development of an enteral nutrition formulary for the hospital. This activity entailed selection of a single enteral product for each major patient condition (e.g., fluid-restricted formula, high protein needs formula, diabetic enteral product, renal failure formula, malabsorption enteral formula, *etc.*) and elimination of duplicative enteral products. Development of an enteral nutrition formulary resulted in an approximate 30% reduction in the budget for purchasing of enteral nutrition products.

Quality improvement initiatives: Numerous quality improvement practice initiatives have been implemented by all trainees following their completion of the program. Examples of these programs include centralization of the storage of intravenous potassium and phosphate products, engagement in glycemic control techniques for diabetic patients requiring nutrition therapy, development of a protocol for the treatment of hypokalemia with intravenous potassium and interdisciplinary collaboration for the development of procedures for pre-operative nutritional assessment and nutritional screening at hospital admission.

Educational responsibilities: Expansion in experiential educational activities among the former trainees were diverse depending on the opportunities available at their institution. All former trainees are currently engaged in experiential teaching of pharmacy students. Two former trainees with pharmacy residents at their institution are actively engaged in clinical nutrition support training of pharmacy residents, whereas one former trainee has extended her involvement beyond the institution’s pharmacy residents to include other visiting hospital pharmacy residents, a pharmacist from another foreign country and medical residents in anesthesiology and endocrinology from her parent institution. Numerous lectures to medical, nursing and pharmacy staff at their parent institutions regarding various aspects of parenteral or enteral nutrition therapy have also been conducted by the former trainees.

Scholarship and organization involvement: One former trainee has written a book chapter designed for pharmacists and nurses regarding enteral nutrition therapy in the hospitalized setting. Another trainee has described her involvement in a multicenter study examining parenteral nutrition safety issues and complications associated with parenteral nutrition. Two trainees have reported the provision of invited presentations regarding nutrition therapy at national and European pharmacy and nutrition congresses.

## 4. Summary

As international pharmacy education and post-education residency training continues to develop, some countries have expanded the role of the pharmacist to include advanced clinical pharmacy services. As a result, there is an increased need for formalized advanced clinical training. It is anticipated that opportunities for clinically-skilled pharmacists will increase as parenteral and enteral nutrition therapy and pharmaconutrition become more complex. This paper describes a unique clinical training program between the University of Tennessee, College of Pharmacy, Regional One Health and nutrition support pharmacists from Spain. Early evidence indicates that those individuals successfully completing this program contributed to the advancement of pharmacy nutrition support practice at their own institution, in Spain and internationally.
